# A Comprehensive and Modality Diverse Cervical Spine and Back Musculoskeletal Physical Exam Curriculum for Medical Students

**DOI:** 10.21980/J8RQ0N

**Published:** 2025-07-31

**Authors:** Konnor Davis, Aaron Frank, Trinidad Alcala-Arcos, Claire Godenzi, Melissa Allison, Clara Riggle, Sangeeta Sakaria, Ariana M Nelson, Alisa Wray, Brian Y Kim

**Affiliations:** 1 University of California, Irvine, School of Medicine, Irvine, CA; 2 University of California, Davis, Department of Emergency Medicine, Sacramento, CA; 3 Stanford University, Department of Internal Medicine, Stanford, CA; 4 University of Washington, Department of Psychiatry, Seattle, CA; 5 University of California, Davis, Department of Anesthesiology and Pain Medicine, Sacramento, CA; 6 Department of Psychiatry, University of California, Irvine, Health, Orange, CA; 7 Cambridge Health Alliance/Harvard Medical School, Boston, MA; 8 University of California, Irvine Health, Department of Anesthesiology and Pain Medicine, Orange, CA; 9 University of California, Irvine Health, Department of Emergency Medicine, Orange, CA; 10 University of California, Irvine Health, Department of Family Medicine, Orange, CA

## Abstract

**Audience:**

The target audience for this small group session focused on the cervical spine and back musculoskeletal physical exam is medical students of all levels, although it is most useful for those early in their career such as first- and second-year medical students. These videos can also be used for other health care professionals such as physicians, physician associates, nurses, or nurse practitioners learning or refreshing their physical exam skills.

**Introduction:**

The physical exam (PE) is one of the core components of a physician’s toolkit and learning to perform the neck and back exam is important. When done correctly, physical exams are a useful tool in evaluating patients and in creating a differential diagnosis. This is especially true for many patient concerns such as neck and back pain and in specialties such as neurology where diagnoses may be established using empiric observation by trained clinicians.^1^

Beginning in 2019, the University of California, Irvine, School of Medicine (UCISOM) revised the physical exam portion of the Clinical Foundations (CF; “doctoring” course) which serves as a four-year longitudinal course for UCISOM students to learn, practice, and improve their history-taking, physical exam, differential diagnosis, and physicianship skills. Focusing on the PE component of the curriculum, a team of physicians reviewed the materials utilized to teach all PE sessions, and these videos specifically, the cervical spine and back musculoskeletal PE for the first- and second-year students. These materials included book chapters and third-party videos; additionally, student and physician feedback were reviewed. Previous student feedback felt that the third-party videos were not engaging and too long (run length per video was upwards of 60 minutes), and students requested videos with slightly more detail for future clinical exams. Utilizing the UCISOM clinical faculty and standardized patients (SP), a team of physician educators and students researched PE best practices for the cervical spine and back musculoskeletal physical exam and developed new video scripts and slides, and ultimately filmed, edited, and produced a series of eight videos demonstrating the cervical and back musculoskeletal PE maneuvers. These videos were one part of a series of fifty-six PE videos developed for learners of a comprehensive physical exam. Other portions of the series focus on vital signs, the cardiovascular exam, pulmonary exam, gastrointestinal exam, neurological exam, head, eyes, ears, nose and throat, and upper and lower extremity exams.

**Educational Objectives:**

By the end of this session, students will be able to: 1) demonstrate how to properly perform a cervical spine and back physical exam, 2) understand the reasoning behind cervical spine and back PE maneuvers, 3) identify the proper technique and equipment to use for the cervical spine and back PE, 4) understand normal and abnormal findings in the cervical spine and back PE, 5) accurately record and report exam findings for the cervical spine and back PE.

**Educational Methods:**

The first-year medical student physical exam small group sessions used a flipped classroom model with the videos serving as a learning resource center (LRC) followed by an in-person, hands-on PE session with standardized patients (SP) led by a Dean’s Scholar.

Prior to the in-person session, the students were required to watch, at minimum, the English cervical spine and back “Full Video” physical exam. Bilingual Spanish speaking students in the UCISOM PRIME-LC (Program in Medical Education for the Latino Community) cohort were also encouraged to watch the Spanish “Full Videos” because their hands-on sessions are completed in Spanish. Learners were also given the option to read about the physical exam, its purpose and steps to perform each maneuver via the Bates Guide to Physical Examination and History Taking which is available via the UCISOM Library.

The in-person, hands-on cervical spine and back musculoskeletal PE sessions occurred in a large group setting with approximately fifty students and four to six faculty members present; students were separated into groups of three to six students at a table with a standardized patient, and the session occurred over the span of two hours. When students arrived at the PE didactic session, the cervical spine and back musculoskeletal exam was discussed, and then groups would practice the PE with the facilitator and standardized patient using the video to guide the examination.

**Research Methods:**

After completion of the small group session, learners from the first- and second-year medical school classes were encouraged to complete a Qualtrics survey regarding the videos and small group sessions. The survey asked students if they felt the videos were helpful, whether they made them more confident at performing physical exams, and if they had high production quality.

**Results:**

As of January 2023, the musculoskeletal cervical spine and back videos received 372 views and downloads, 318 unique viewers, and delivered 1,776 minutes of content. Thirty-one learners (response rate of approximately 25%) responded to the survey. The educational quality of all the musculoskeletal videos, including cervical spine and back, averaged 4.71 out of 5, the usefulness averaged 4.63 out of 5, and the production quality averaged 4.56 out of 5.

**Discussion:**

Based on the results from our survey as well as end-of-course feedback and verbal feedback sessions with leaners, we deem this educational content efficacious. The videos and all content related to them (eg, scripts, graphics, voice over) were assessed by a team of seven physicians. We feel the efficacy of the videos increases when implemented with other modalities of LRC such as written material or podcasts. Using multiple other modalities allows learners to experiment and utilize the modality that will aid their learning the best. Importantly, we also learned from the implementation of the videos that auto-generated subtitles for the videos in Spanish were often incorrect. This was in part due to the learning management system used to maintain the videos, but also could have been alleviated by including subtitles within the videos themselves. Overall, video content demonstrating these maneuvers, both with and without additional graphics and voice-over, and in two languages, was highly efficacious for UCISOM students in the CF course.

**Topics:**

Physical exam, cervical spine, neck, back, low back, musculoskeletal, video, voice-over, medical student education.

## USER GUIDE

**Table:** 

List of Resources:	
Abstract	1
User Guide	3
Small Groups Learning Materials Video Links	7

**Learner Audience:**

Medical Students

**Time Required for Implementation:**

Cervical Spine Full Video (English) – 6 min

Back Full Video (English) – 7 min

Cervical Spine Exam Only (English) – 5 min

Back Exam Only (English) – 5 min

Cervical Spine Full Video (Spanish) – 6 min

Back Full Video (Spanish) – 7 min

Cervical Spine Exam Only (Spanish) – 4 min

Back Exam Only (Spanish) – 5 min

Small Group Hands-On Session – 30 to 120 minutes.

**Recommended number of learners per instructor:**

For these small groups, we recommend three to six students to one instructor.

**Topics:**

Physical exam, cervical spine, neck, back, low back, musculoskeletal, video, voice-over, medical student education.

**Objectives:**

By the end of this session, students will be able to:

1. Demonstrate how to properly perform a cervical spine and back physical exam
2. Understand the reasoning behind cervical spine and back PE maneuvers
3. Identify the proper technique and equipment to use for the cervical spine and back PE
4. Understand normal and abnormal findings in the cervical spine and back PE
5. Accurately record and report exam findings for the cervical spine and back PE

### Linked objectives, methods and results

Video delivery of educational content, specific to medical students, has been associated with higher student satisfaction and a score improvement compared to only using textbook learning.^2^ For this session, we desired to have learners engage with the content using multiple modalities while also allowing them to interact with the content on their own schedule. To achieve these goals, learners were provided with the material in three modalities: visual (prerecorded video content and preselected readings, available 24/7), auditory (prerecorded video content, available 24/7), and tactile (group sessions with a physician leader). By the start of the in-person session, learners will have been exposed to all major information necessary to perform and understand the exam (objectives 1–5). During the session, a basic understanding of the anatomy, physiology, and clinical reasoning for preforming the exam was assumed in order to focus on tactile skills such as where to palpate on the patient’s spine (objectives 1, 3).

Learners had ample time to learn and review the preparatory content while also having two hours of guided, hands-on, in-person practice. Supplemental practice sessions were made available throughout the academic year, and all video content and readings are readily accessible throughout a learner’s time at the institution.

Visual, auditory, read and write, and kinesthetic (VARK) is the main educational framework that guides this learning session.^3^ In VARK, an individual’s preferred method of gathering, processing, interpreting, organizing, and analyzing information is ascertained and profiled. In the learning stage, we provide the materials and time to allow for learners to use their preferred method of gathering and processing (objectives 1 and 2). In the in-person practice stage, we provide a group environment well equipped to practice the cervical and spine and back musculoskeletal examination maneuvers and interpret exam findings (objectives 3, 4, and 5). This will ultimately provide the fundamentals to prepare learners to organize an assessment and plan and analyze pertinent positives and negatives leading to a differential diagnosis later in their medical education.

Videos were filmed in 4K quality with dedicated audio and two, or more, camera angles. For each cervical spine and back musculoskeletal physical exam, four videos were produced: “Exam Only” videos include the physician performing the exam with background music, while “Full Videos” include the exam with explanation slides, graphics, and voiceovers to increase the instructional value; both types of videos were produced in English and Spanish. Video content, especially that which includes voice-overs, slides, and graphics, can present information in a variety of ways to accommodate students’ learning preferences (eg, auditory, visual, written).^$^ All four videos per PE were uploaded to students’ learning management system as part of the pre-learning LRC for students to review the PE maneuvers prior to in-person training sessions. These videos allow medical students increased flexibility by providing them the ability to review material at their own pace.^5^ The 2022–2023 academic year was the first year where all videos were provided to students at the UCISOM.

The efficacy of the educational content was first approved by the clinical faculty at UCI, and then a small batch of videos were initially released to test their effectiveness (through student feedback) before continuing to produce the rest of the videos. Student and faculty narrative feedback was taken into consideration prior to the final release of the videos. All videos were included for first-, second-, and third-year students to utilize as review prior to other SP sessions, objective structured clinical examinations (OSCEs), or patient care.

### Recommended pre-reading for instructor

- Bates Guide to Physical Examination and History Taking (13e), Chapter 24: Musculoskeletal System.
- Cervical Spine Full Video (English) or Cervical Spine Full Video (Spanish) and Back Full Video (English) or Back Full Video (Spanish).

### Learner responsible content (LRC)

- Bates Guide to Physical Examination and History Taking (13e), Chapter 24: Musculoskeletal System.
- Cervical Spine Full Video (English), Back Full Video (English), Cervical Spine Exam Only (English), Back Exam Only (English) or Cervical Spine Full Video (Spanish), Back Full Video (Spanish), Cervical Spine Exam Only (Spanish), Back Exam Only (Spanish).

### The following is the list of materials you will need

- Clinical skills room or any room with
  ○ Exam table or regular table for patient to sit on
  ○ Yoga or massage mat
  ○ Sheets for draping
- Computer and monitor/projector with audio to play exam video
- Standardized patient or students consenting to examine each other

### Session Setup

- If in small group rooms, student groups of three to six learners + one faculty member
- If in large group room, students in groups of two to four + several faculty members walking around the room to assist during the exam.
- We recommend having the video available during the session so that it can be played in the background to help guide the learners.
- Start by having the instructor show the entire exam; then have the instructor walk through the basics of each maneuver as demonstrated in the videos.
- Then allow learners the opportunity to practice the maneuvers on the SP while using the PE videos on their electronic devices, or playing on a large monitor in the room.
  ○ Option 1: One learner performs the entirety of the exam while the other students, SP, and instructor watch and provide feedback and corrections. Then the next learner performs the entirety of the exam.
  ○ Option 2: Demonstrate one section of the exam; then have each learner perform that section of the exam before moving on to demonstrate the next section of the exam and have each learner perform the next section. Continue until the entire exam has been demonstrated and practiced. Then have each learner perform the exam in its entirety. Instructors and SPs provided feedback and corrections during each section of the exam.

### Results and tips for successful implementation

#### Results

As of January 2023, the entire project (56 videos) had garnered the following statistics: 3,568 views and downloads, 1,122 unique viewers, and 26,513 minutes of content delivered. Eighty-six learners responded to the survey and 84 found the videos helpful (98%), 80 found that the videos made them more confident at performing physical exams (93%), and 80 felt the videos had high production quality (93%). For the project, the educational quality of the videos averaged between 4.65–4.72 out of 5 (1-not educational, 3-neutral, 5-very educational), the usefulness averaged between 4.56–4.59 out of 5 (1-not useful at all, 3-neutral, 5-very useful), and the production quality averaged between 4.63–4.73 out of 5 (1-poor quality, 3-neutral, 5-excellent quality).

Specific to the musculoskeletal cervical spine and back videos, there were 372 views and downloads, 318 unique viewers, and 1,776 minutes of content delivered. The educational quality of all the musculoskeletal videos, including cervical spine and back, averaged 4.71 out of 5 (n=31), the usefulness averaged 4.63 out of 5 (n=31), and the production quality averaged 4.56 out of 5 (n=31).

A per-video breakdown and overall total can be found below:

**Table:** 

Cervical Spine	Full Video (English)	Exam Only (English)	Full Video (Spanish)	Exam Only (Spanish)	Total
Views and Downloads	50	7	0	0	57
Unique Viewers	38	6	0	0	42
Minutes Delivered	197	14	0	0	211

**Table:** 

Back	Full Video (English)	Exam Only (English)	Full Video (Spanish)	Exam Only (Spanish)	Total
Views and Downloads	216	62	23	14	315
Unique Viewers	183	60	18	13	274
Minutes Delivered	1233	197	89	46	1565

#### Tips for Successful Implementation

Learners, instructors, and standardized patients should be provided with the PE videos at least one week in advance to ensure appropriate time to review materials prior to the session. For the in-person session, we recommend small groups of three to six learners with an instructor and an SP, although for the cervical spine and back musculoskeletal exam, students, if comfortable, can practice with each other as well. We recommend holding the training session in a clinical skills center or clinical space with appropriate exam table, lights, and privacy; however, if this is not available, having a table with padding for the SP and sheets for draping is recommended. If SPs are not utilized, then we recommend that learners wear “gym” style clothing and use sheets for draping to assess each other. It is useful to have a computer, tablet, or TV to play the video in the room to assist with the exam.

The cervical spine and back musculoskeletal back exam group session lasted about two hours. During the initial portion of the session, the instructors walked through the basics of each maneuver and demonstrated the exam as was shown in the videos. Then the learners had the opportunity to practice the maneuvers on the SP while utilizing the PE videos on their electronic devices, or playing on a large monitor in the room, to assist as needed. Groups were given the option of learners performing the entirety of the exam each while watching each other or having the learners each performing a section of the exam one after another before moving on to the next portion of the exam. Each group was allowed to determine what worked best for their group, although most groups preferred to break the exam down into sections and then each perform the exam in its entirety at the end. Instructors and SPs provided feedback and corrections during the exam.

### Video Links

- Cervical Spine Full Video (English) – 6 min
  ○ https://youtu.be/CUYZ_gJUugU
- Back Full Video (English) – 7 min
  ○ https://youtu.be/ctCOgf_Ck4w
- Cervical Spine Exam Only (English) – 5 min
  ○ https://youtu.be/DWv4p3GrYG8
- Back Exam Only (English) – 5 min
  ○ https://youtu.be/yloBli55iPo
- Cervical Spine Full Video (Spanish) – 6 min
  ○ https://youtu.be/KbOCNYfE0X8
- Back Full Video (Spanish) – 7 min
  ○ https://youtu.be/EqNI9w0Blxg
- Cervical Spine Exam Only (Spanish) – 4 min
  ○ https://youtu.be/Ee29axnHRzs
- Back Exam Only (Spanish) – 5 min
  ○ https://youtu.be/UmAT0ILYcWY

## Figures and Tables

**Figure:**
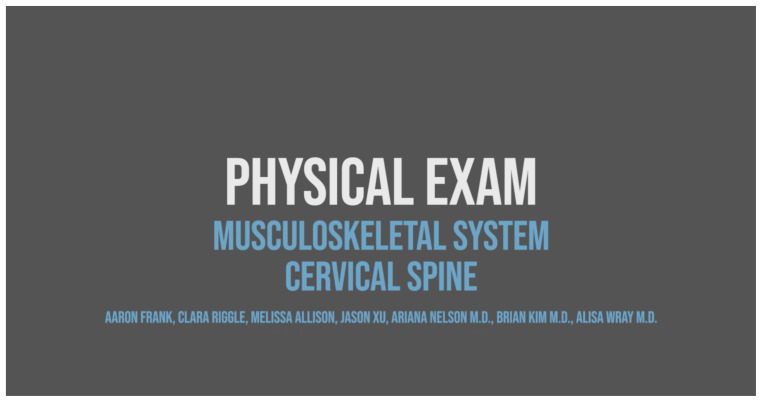
Video Links: Cervical Spine Full Video (English) – https://youtu.be/CUYZ_gJUugU Cervical Spine Full Video (Spanish) – https://youtu.be/KbOCNYfE0X8 Cervical Spine Exam Only (English) – https://youtu.be/DWv4p3GrYG8 Cervical Spine Exam Only (Spanish) – https://youtu.be/Ee29axnHRzs

**Figure:**
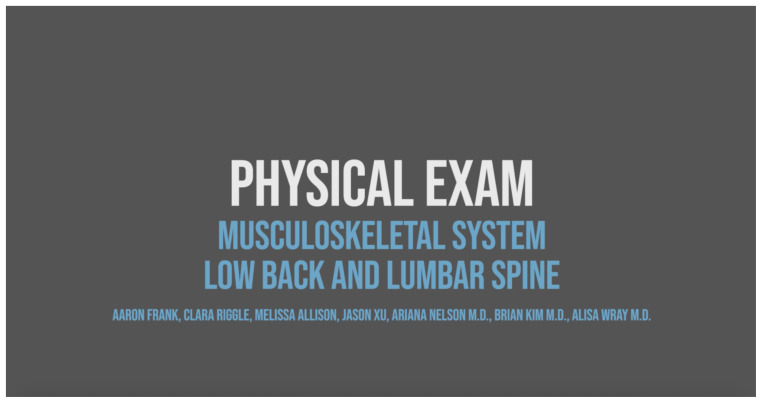
Video Links: Back Full Video (English) – https://youtu.be/ctCOgf_Ck4w Back Full Video (Spanish) – https://youtu.be/EqNI9w0Blxg Back Exam Only (English) – https://youtu.be/yloBli55iPo Back Exam Only (Spanish) – https://youtu.be/UmAT0ILYcWY
